# Supplementary figures and images for: Miltefosine enhances infectivity of a miltefosine-resistant Leishmania infantum strain by attenuating its innate immune recognition
Source: PLoS Negl Trop Dis. 2021 Jul 22;15(7):e0009622. doi: 10.1371/journal.pntd.0009622 (PMC8330912; doi:10.1371/journal.pntd.0009622)

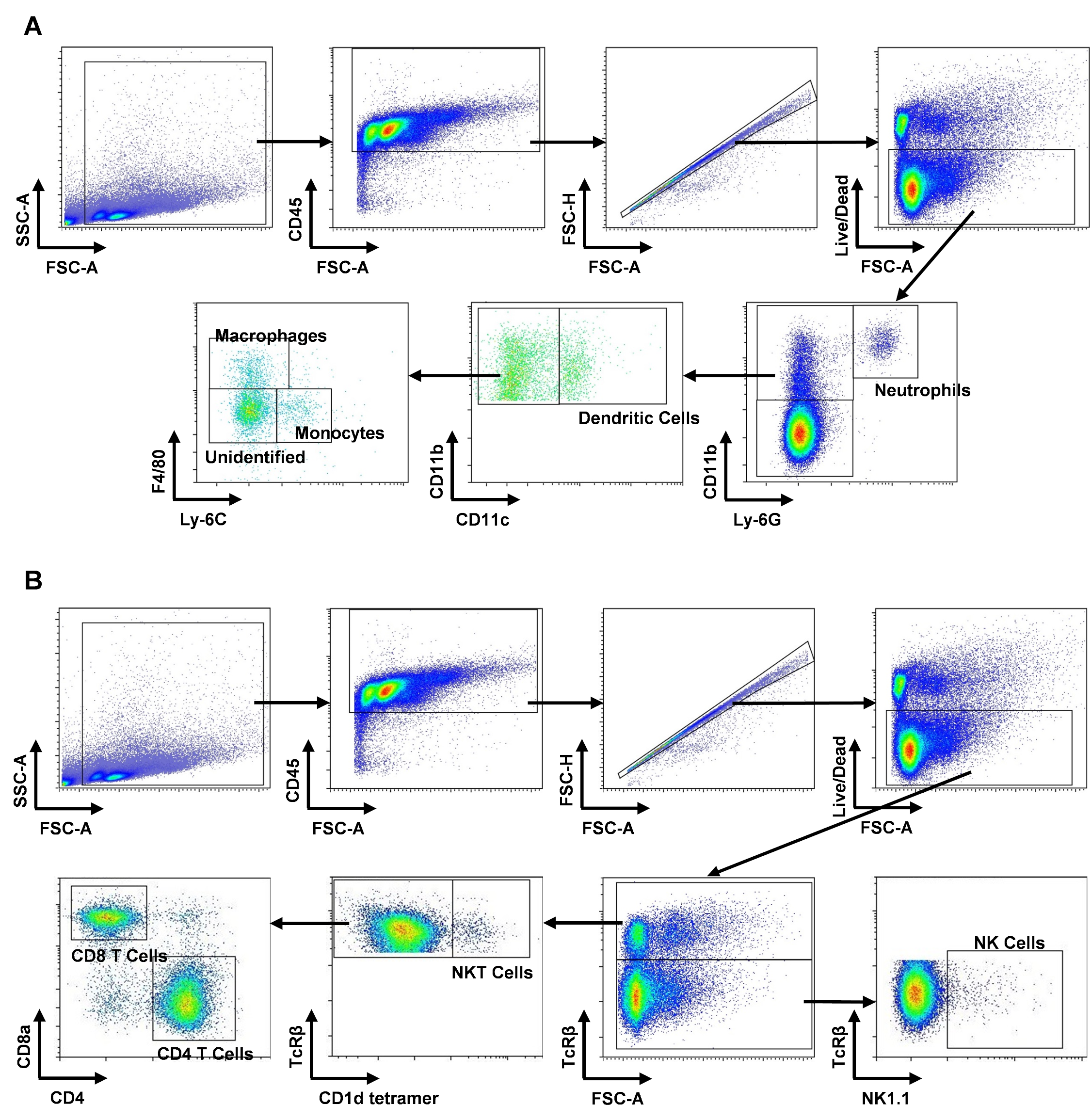

Supplement: S1 Fig — (A) Live CD45+ singlet cells were characterized as being CD11b+, a general myeloid marker. CD11b+ cells were further characterized as neutrophils (Ly-6G+), dendritic cells (CD11c+), monocytes (Ly-6C+ F4/80-), macrophages (Ly-6Clow F4/80+) and an unidentified CD11b+ population (Ly-6G- CD11c- Ly-6Clow F4/80-). (B) Lymphocytes were characterized as NK cells (TcRβ- NK1.1+), NKT cells (TcRβ+ CD1d tetramer+), CD4+ T cells (TcRβ+ CD1d tetramer- CD4+) and CD8+ T cells (TcRβ+ CD1d tetramer- CD8a+). (TIF) [file pntd.0009622.s002.tif]

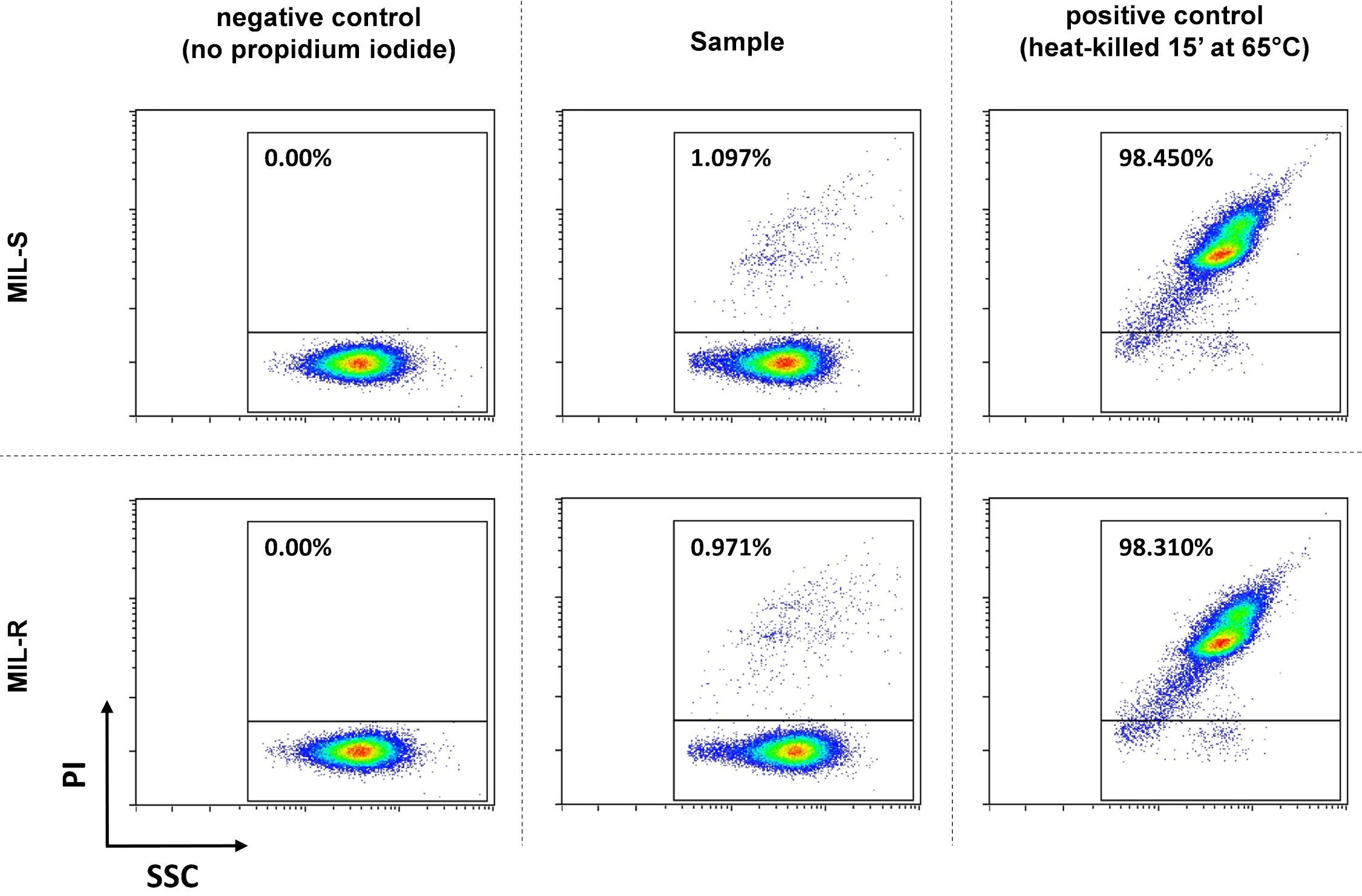

Supplement: S2 Fig — Propidium iodide live/dead staining of stationary phase MIL-SPpyRE9/DsRed and MIL-RPpyRE9/DsRed infection inoculums, revealing no differences between both parasite strains. Unstained parasites were used as negative controls whereas heat-killed parasites (15’ at 65°C) served as positive controls. A representative plot of each condition is shown. Experiments were carried out in triplicate with two to three technical replicates per condition. Percentages shown are the average percentage of dead parasites for each condition. (TIF) [file pntd.0009622.s003.tif]

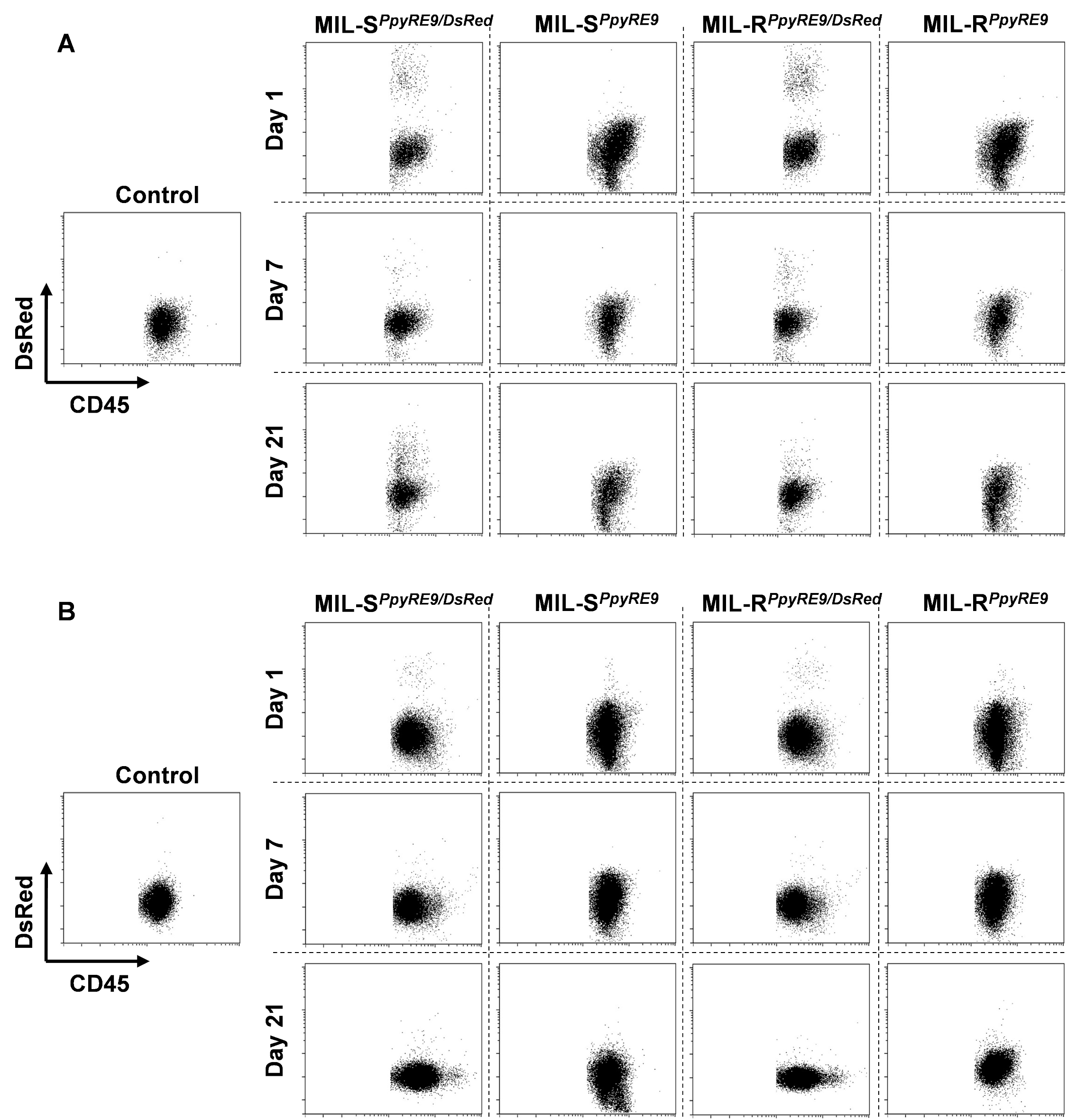

Supplement: S3 Fig — In order to validate that the observed DsRed signals in the CD45+ liver cells corresponds to parasite-infected cells, MIL-SPpyRE9/DsRed and MIL-RPpyRE9/DsRed infected liver (A) and spleen cells (B) were compared to uninfected controls and MIL-SPpyRE9 and MIL-RPpyRE9 infected controls at 1 dpi, 7 dpi and 21 dpi. A representative plot of each condition with equal number of events is shown. (TIF) [file pntd.0009622.s004.tif]

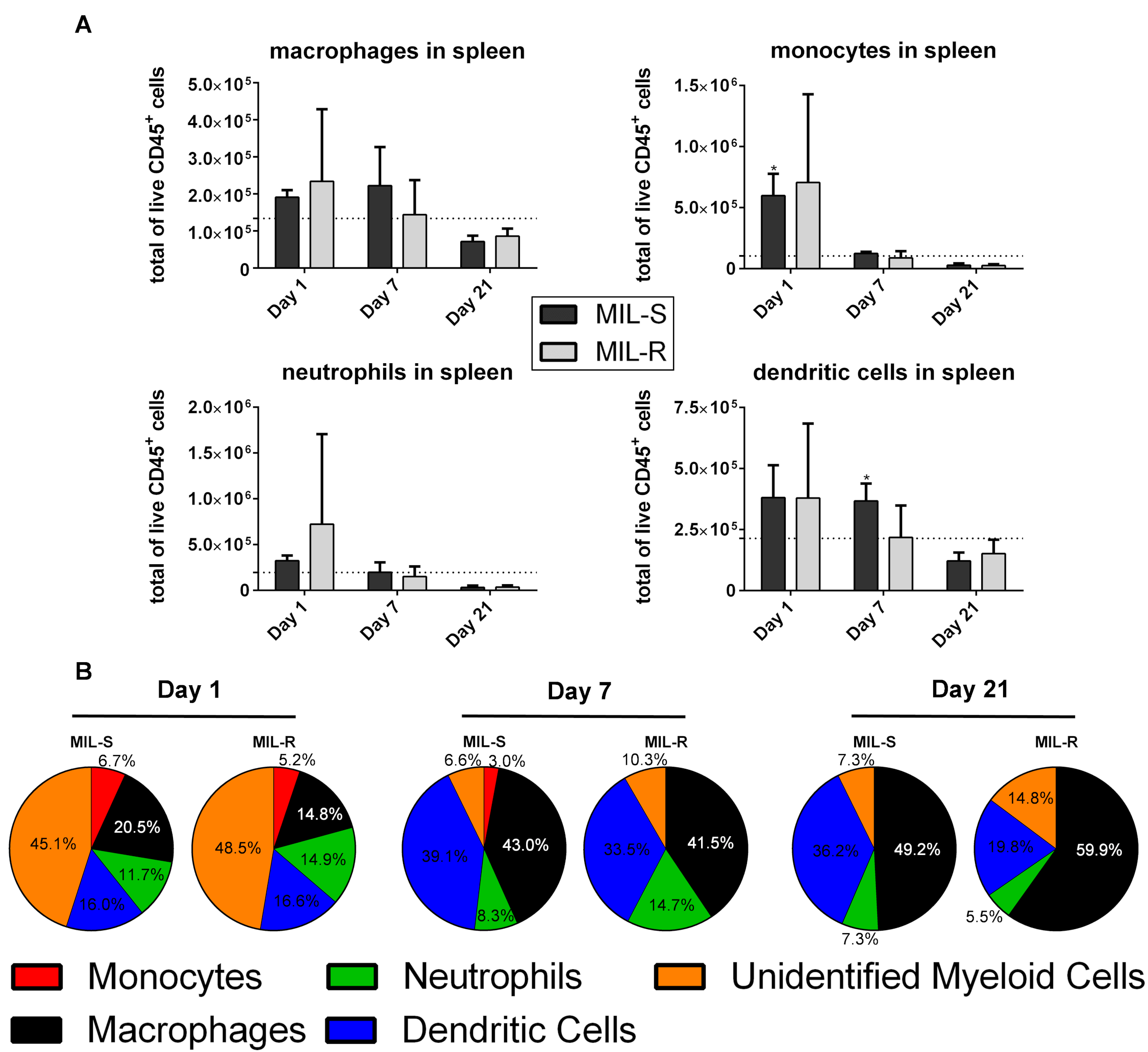

Supplement: S4 Fig — (A) Total number of macrophages, monocytes, neutrophils and dendritic cells in the spleen of C57Bl/6 mice infected with MIL-SPpyRE9/DsRed and MIL-RPpyRE9/DsRed at 1, 7 and 21 dpi. Dotted lines indicate the average number of cells in naive animals. (B) Distribution of infected cells among the CD11b+ myeloid compartment in the spleen of MIL-SPpyRE9/DsRed and MIL-RPpyRE9/DsRed infected C57Bl/6 mice at 1, 7 and 21 dpi. Experiments were carried out in triplicate with 3 to 5 mice per infection group and three independent repeats were performed. Results are expressed as mean ± SD (* p≤0.05). (TIF) [file pntd.0009622.s005.tif]

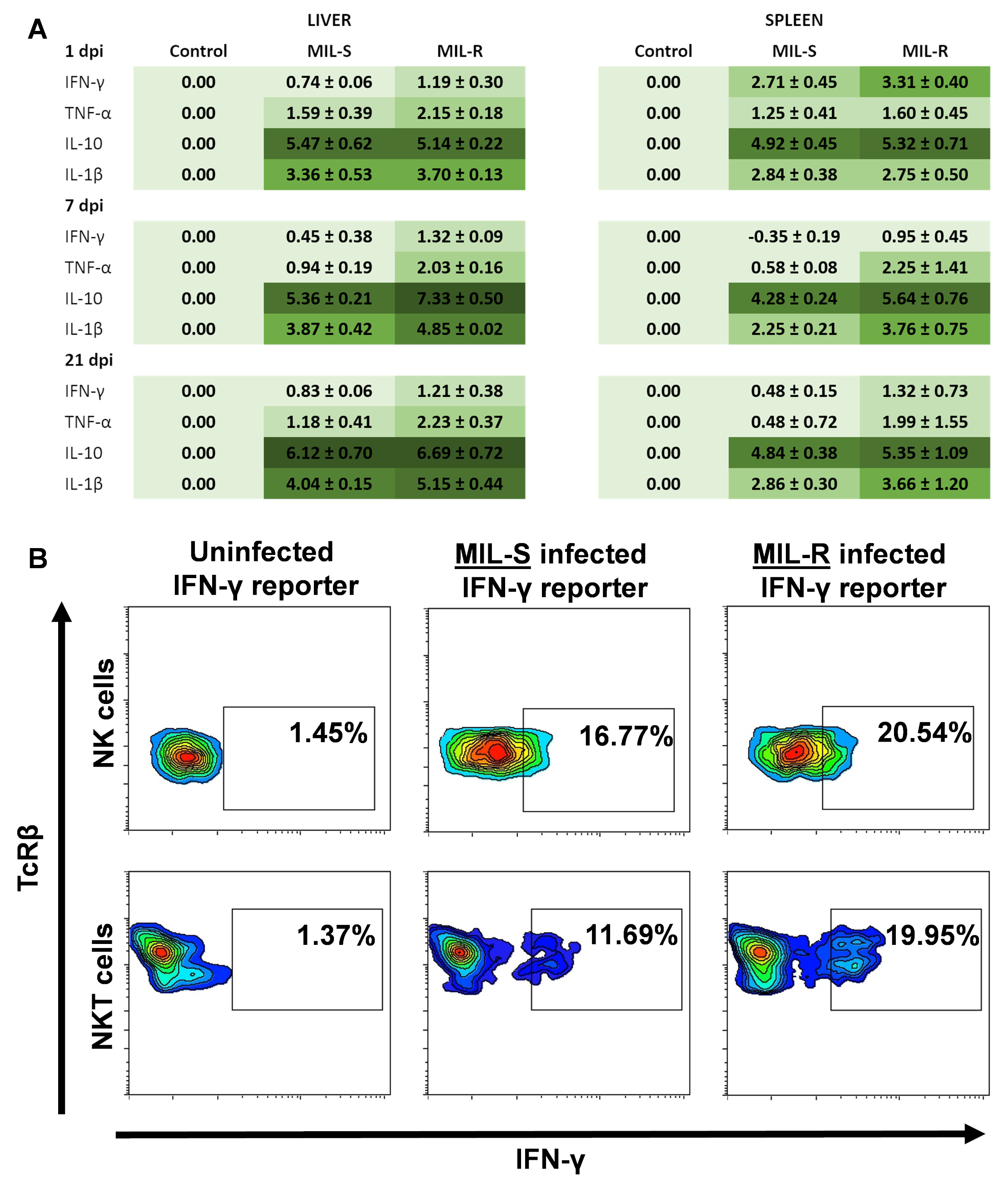

Supplement: S5 Fig — (A) Heat map representing the transcription of inflammatory genes in the liver and spleen of MIL-SPpyRE9/DsRed and MIL-RPpyRE9/DsRed infected C57Bl/6 mice at 1, 7 and 21 dpi. Individual transcript levels were normalized using the EEF2 reference gene. Results are expressed as the mean log2-fold change ± SD and are based on 1 repeat with 3 mice per infection group and 3 control mice. (B) Contour-density plots of liver NK (top panel) and NKT (lower panel) cells of uninfected, MIL-SPpyRE9/DsRed and MIL-RPpyRE9/DsRed infected IFN-γ reporter mice: a representative plot of each infection group is shown. Experiments were carried out in duplicate with 3 mice in each infection group. Results are expressed as mean ± SD. (TIF) [file pntd.0009622.s006.tif]

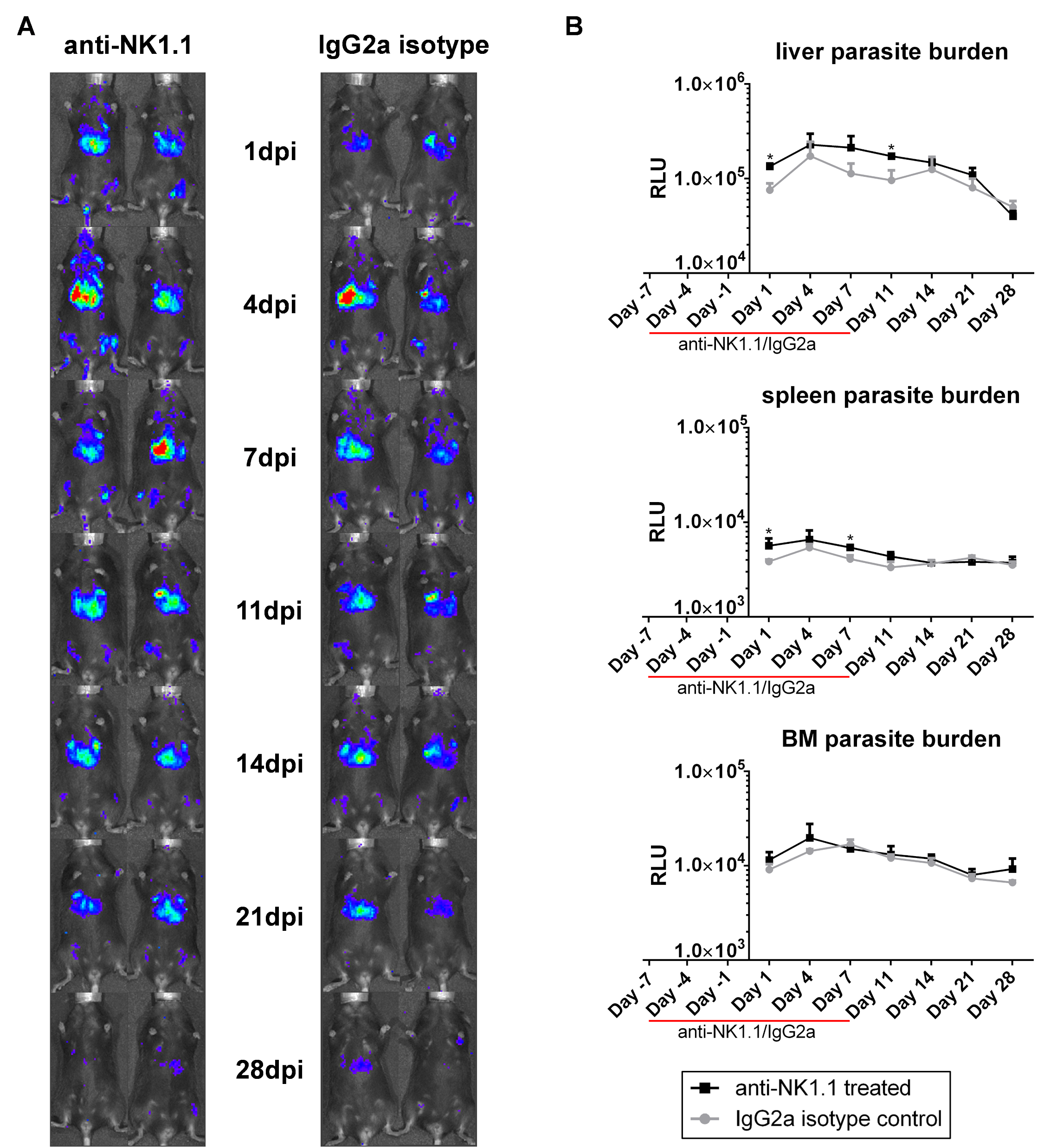

Supplement: S6 Fig — (A) Bioluminescent imaging using an exposure time of 15 min of MIL-RPpyRE9/DsRed infected C57Bl/6 mice treated with an anti-NK1.1 depleting antibody or an IgG2a isotype control antibody for 2 weeks starting 1 week before infection. (B) Mean RLU values of liver, spleen and BM bioluminescent signals during the first 4 weeks of MIL-RPpyRE9/DsRed infection in anti-NK1.1- and IgG2a isotype-treated C57Bl/6 mice. Experiments were carried out in duplicate with 3 mice in each infection group. Results are expressed as mean ± SD (* p≤0.05). (TIF) [file pntd.0009622.s007.tif]

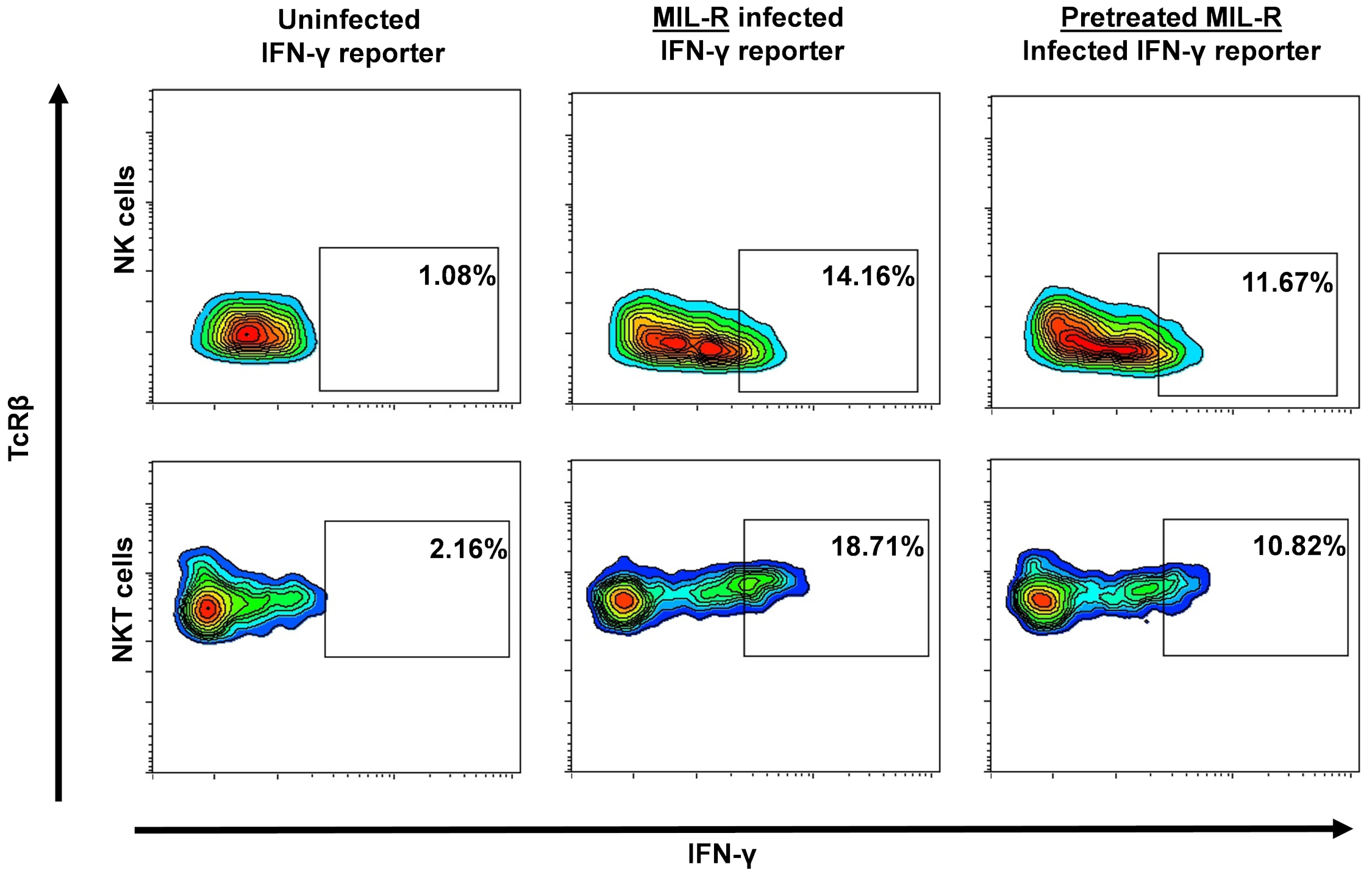

Supplement: S7 Fig — Contour-density plots of liver NK cells (top panel) and NKT (lower panel) of uninfected, MIL-pretreated MIL-RPpyRE9/DsRed and MIL-RPpyRE9/DsRed infected IFN-γ reporter mice at 1 dpi. A representative plot of each infection group is shown. Experiments were carried out in duplicate with 3 mice in each infection group. (TIF) [file pntd.0009622.s008.tif]
